# Glioblastoma Multiforme Stem Cells

**DOI:** 10.1100/tsw.2011.42

**Published:** 2011-04-19

**Authors:** Irena Dimov, Deasanka Tasić-Dimov, Irena Conić, Vladisav Stefanovic

**Affiliations:** University of Niš, School of Medicine, Niš, Serbia

**Keywords:** glioblastoma multiforme, tumor stem cell, markers, epigenetics, immunosuppression, therapy

## Abstract

Glioblastoma multiforme (GBM) is an aggressive, malignant, and lethal brain tumor, resistant to all current forms of treatment. The rapidly emerging focus on cancer stem cells embodies a paradigm shift in our understanding of tumor pathogenesis, while the development of powerful genome-wide screening techniques has provided cause for optimism related to the development of more reliable therapies primarily targeting GBM stem cells (GBMSCs). There are promising mounting data on providing new molecular targets and predictive markers of response, leading to more effective therapies of GBM, guided by patient-specific genetic and epigenetic profiling. However, the achievement of efficient GBMSC targeting also requires an adequate understanding of the unique microenvironment, and the relationship with the immune system in the central nervous system (CNS) and CNS tumors. The endogenous immune regulation is likely to limit or abrogate the efficacy of the host's immune response, as well as the developed immunotherapeutic strategies at present. Therefore, a comprehensive understanding of the mechanisms underlying the GBM-induced immunosuppression is indispensable. This review presents a summary of the present knowledge both on GBMSCs and the GBM, and/or GBMSC-related mechanisms of developing both local and systemic immunosuppression, of which an understanding may lead to the development of the novel and effective therapeutic strategies.

## INTRODUCTION

Glioblastoma multiforme (GBM) or WHO Grade IV malignant glioma is a deadly malignant brain tumor, resistant to all current forms of treatment. It may develop *de novo* (primary GBM) or as the result of the malignant progression from a low-grade glioma WHO grade II and III (secondary GBM). Patients with GBM have a median survival of approximately 15 months. The immune system in GBM tumor patients has been shown to be in a highly suppressed state, making it difficult to elicit antitumor responses. GBM are highly recurrent even after surgery, chemotherapy, radiation, and immunotherapy, whether they rise *de novo* or develop as the secondary GBM. In the last decades, the treatment strategies for gliomas have not changed appreciably because of the limited understanding of the biology of the disease. The rapidly emerging focus on cancer stem cells embodies a paradigm shift in our understanding of tumor pathogenesis.

GBMs are heterogeneous tumors, histopathologically, radiographically, and genetically. Current literature delineates four subtypes of GBMs. Ninety-seven percent of the tumors are defined as the "classic" subtype, which carries extra copies of the *EGFR* and overexpressed EGFR, whereas the gene *P53* is rarely mutated[1]. In contrast, the proneural subtype often has high rates of alterations in *P53*, *PDGFRA*, and *IDHl* genes. The mesenchymal subtype is characterized by high rates of mutations or other alterations in *NF1*, the gene encoding neurofibromatosis type 1, fewer alterations in the *EGFR*, and a lower expression of EGFR compared to other types[2]. Secondary glioblastomas are the most rare and are more common in younger patients (mean age: 45 vs. 62 years)[3]. The tumor may take on a variety of appearances depending on the amount of hemorrhage, necrosis, or its age.

GBMs, much like other malignomas, are composed of a heterogeneous mix of neoplastic and non-neoplastic cells that include both native and recruited cells. The GBM stem cells (GBMSCs) can be defined as a slow-cycling, but highly tumorigenic, GBM cell subset that is selectively capable of indefinite self-renewal and differentiation to bulk populations of nontumorigenic cancer cells. These two basic properties make the GBMSC population the prime candidate for tumor maintenance and recurrence. They are also implicated in the development of chemo- and radioresistance[4,5,6,7,8,9].

The current therapy for GBM is typically based on cytoreductive strategies, which preferentially target the bulk of the tumor mass and possibly exempt some of the GBMCSs. The achievement of specific tumor stem cell targeting holds some promise for future effective GBM therapy, but also requires an adequate understanding of the unique microenvironment in the central nervous system (CNS) and CNS tumors. Critical to the maintenance of the stem cells in the adult brain are microenvironmental cues and cell-cell interactions that act to balance stem cell quiescence with proliferation, and to direct neurogenesis vs. gliogenesis lineage decisions. Histologic and *ex vivo* cell culture studies of mammalian tissues showed that neural stem cells (NSCs) lie within a vascular niche that regulates stem cell self-renewal. Subventricular zone (SVZ) stem cells populate a complex niche adjacent to the lateral ventricles, whose components are just beginning to be uncovered[10,11,12,13]. This continual supply of new neurons and glia then provides the postnatal and adult brain with an added capacity for cellular plasticity, albeit one that is restricted to a few specific zones within the brain. Atypical ectopic perivascular niches were recently described around perivascular areas of the inflamed CNS, suggesting their potential role in the pathogenesis of several CNS diseases[14]. Therefore, GBMSCs might involve an intrinsic mutation of the stem and/or progenitor pool, as well as the deregulation of the niche. Furthermore, the molecular machinery used by normal stem cells may be "hijacked" by the GBMSCs for proliferation and invasion[13,15,16]. However, the exact identity and cells of origin of GBMSCs remain elusive, as well as events initiating their development and maintenance.

For many years, the CNS has been postulated as an "immunoprivileged" compartment with no direct links with the immune system. Current data do not only support the presence of resident CNS macrophages/microglia, but also suggest the active interaction of the CNS with peripheral immune cells. In contrast to other organs, in the CNS, dendritic cells are not thought to be present in normal parenchymal tissue or perivascular space, although their presence is shown in the meninges and choroids plexus. Dendritic cells from cerebrospinal fluid have been found to migrate to B-cell follicles of cervical lymph nodes[17,18,19]. Immunotherapy strategies targeting GBMs, such as dendritic cell vaccines or the immunogene transfection, have shown dramatic results in *in vitro* studies and some animal models[20,21]. However, they have not yet been translated into effective clinical practice and the GBM-mediated immunosuppression might be a critical factor. Furthermore, a large number of observations suggest that certain types of tumor microenvironmental immune cells are not innocent bystanders, and that they actively affect tumor development and progression. This review summarizes the present knowledge both on GBMSCs and the immune responses in GBM patients in an attempt to understand the link between the biology of the tumor evolution and consequential systemic effects. Particularly, we are going to focus on the mechanisms underlying local and systemic immunosuppression in an attempt to understand some of the major current obstacles in GBM immunotherapy that, if overcome, could lead to the development of effective immunotherapeutic strategies specifically targeting GBMSCs.

## EVIDENCE FOR GBMSCs

There is growing evidence that adult tissues contain a set of tissue stem cells that might undergo malignant transformations while retaining their stem cell characteristics. Aggressive brain tumors often contain poorly differentiated cells, reflected in the use of the term “blastoma”, as well as in areas of divergent differentiation. There is extensive diversity between the tumor cells, including the different capacity for *in vitro* and *in vivo* growth, a property ultimately linked to the cell’s differentiation status. GBMs are highly invasive and the patterns of glioma cell infiltration (referred to as the secondary structures of Scherer) correspond to the neoplastic cell reacquisition of the primitive migratory behavior during the development of the CNS. These glioma cells migrate through the normal parenchyma, collect tightly below the pial margin (subpial spread), surround neurons and vessels (perineuronal and perivascular satellitosis), and migrate through the white matter tracks (intrafascicular spread)[22].

Several recent reports proposed that normal stem cells and GBMSCs share the expression of several markers, the ability for self-renewal and differentiation, and some signaling pathways involved in the regulation of cellular survival and proliferation[4,5,6,7]. On the other hand, it is well known that the presence of the embryonic stem-like gene expression signatures in human cancers is associated with aggressive histopathology, which supports the clinical significance for the stem-like cellular phenotype.

GBMSCs *in vitro* have an immortal proliferative potential in the presence of the EGF and the bFGF, and closely mirror the phenotype and genotype of primary tumors. Surprisingly, these cells can differentiate into both neuronal and glial lineages when withdrawing these growth factors and adding serum, similar to NSCs[23,24]. The 9L gliosarcoma model in rats and the GL261 glioma model in mice further supported the idea of GBMSC existence[25,26]. It has also been demonstrated that GBMSCs exist in human high-grade gliomas and could be isolated from them[27,28]. As mentioned, the SVZ maintains the ability to generate neurons and glia throughout adulthood, and in animal models, high-grade gliomas can be generated from this cell population. It has been reported that more aggressive patterns of GBM recurrence are associated with tumors that contact the SVZ, and that these tumors may express a ‘‘stem-cell–derived’’ phenotype that could impact treatment decisions. It was suggested that cells responsible for gliomagenesis might be produced in the SVZ, but migrate towards the cortex before tumorigenesis. SVZ-contacting cells overexpress several genes associated with survival of the GBM patients and with the suppression of the immune system[29].

## GBMSC MARKERS

GBMSCs have been characterized mostly on the basis of functional criteria, such as their proliferation as neurospheres when cultured in the presence of growth factors, i.e., “neurosphere assay”. The “neurosphere assay” is an *in vitro* model developed to assign the stem-like cell frequency within the tumor. GBM samples are dissociated and resuspended, obtaining limiting dilution. After primary sphere forming is noted, sphere cells are again dissociated, separately plated, and fed until 7 days, when the percentage of wells not containing novel spheres for each cell-planting density is calculated and plotted against the number of cells per well[4,5]. Current evidence for the existence of prospectively identifiable GBMSCs among cancer bulk populations has been generated using the marker-specific genetic lineage tracking of molecularly defined cancer subpopulations in competitive tumor-development models.

### Nestin

Nestin is an intermediate filament (IF) protein produced in stem cells in the mammalian CNS during development. Formidable evidence indicates that nestin expression highly correlates with “stemness”. It is involved in the organization of the cytoskeleton, cell signaling, organogenesis, and cell metabolism, and it represents the proliferation, migration, and other characteristics of multilineage progenitor cells. NSCs that take on more committed roles decrease the nestin expression and subsequently up-regulate alternative IFs, such as neurofilaments in committed neurons and GFAP in glial precursors[30,31]. The down-regulated nestin may be re-expressed in the adult organism under certain pathological conditions, such as brain injury, ischemia, inflammation, and neoplastic transformation[30,31,32,33].

## CD133/Prominin

CD133/Prominin was originally found on neuroepithelial stem cells in mice. CD133+ cells isolated from the fetal liver and the umbilical cord blood were capable of *in vitro* differentiation to neuronal cells, as well as glial cells, including astrocytes. It is a cell surface marker expressed on normal human NSCs, down-regulated in differentiated cell lines, and expressed in several cancers and acutely dissociated brain tumor cells. Its localization in membrane protrusions suggests an involvement in the mechanisms influencing cell polarity, the migration and interaction of stem cells with the neighboring cells and/or extracellular matrix[34,35,36,37]. CD133 expression showed some prognostic potential in GBM patients[38]. In a recent study, it was proposed that the coexpression of nestin and CD133 was associated with a poor prognosis of malignant gliomas and even correlated better with the clinical course than the histological grading[34]. The coexpression of CD133 and Ki67 was demonstrated as an important prognostic factor of the progression of the disease and a poor clinical outcome[39].

However, the suggestion that only CD133+ cells are capable of self-renewal and recapitulating the parental tumor must be viewed with caution. Wang et al. demonstrated that CD133- cells, isolated from human brain tissue, also have the ability to form tumors when transplanted in mice, and can also give rise to CD133+[40]. Other authors also reported the presence of the CD133- population with GBMSC-like properties[41,42]. These discrepancies may also be the result of experimental differences or variations between or inside brain tumors. Given the complex environmental and other factors affecting the CD133 epitope expression *in vitro*, it may not be surprising that there are some conflicting reports in the literature regarding the existence of CD133- GBMSCs. Indeed, most bona fide *in vivo* stem cells are in a low cycling state, whereas *in vitro* stem cells are highly proliferating. On the other hand, the abnormal and variable microsurroundings charged with a variety of cytokines, growth factors, and oxidative stress indicators might irreversibly alter the genotype and phenotype of initial GBMSCs, giving rise to different subpopulations that might retain stem-like properties. Some studies indicate that the expression of CD133 could be regulated by hypoxia as well as mitochondrial dysfunction (genetic and chemical). This suggests that the expression of CD133 among glioma cells might reflect an environmental stress response[43]. All these data imply that multiple, different cell surface markers are probably necessary to characterize GBMSCs more consistently.

## Musashi-1

Musashi-1 is an RNA-binding protein that is expressed in the SVZ of numerous vertebrates. Similar to nestin, the Musashi-1 expression occurs predominantly in proliferating, multipotent, neural precursor cells and is progressively down-regulated as differentiation proceeds, finally to disappear in terminally postmitotic neurons[44,45]. Musashi-1 inhibits the translation of mRNAs believed to be involved in the differentiation of NSCs and increasing evidence points towards its involvement in tumorigenesis[45]. The level of the Musashi-1 expression has been correlated with the grade and aggressiveness of the malignancy, as well as the mitotic activity in brain tumors[46]. *In vitro* neurospheres derived from brain tumors showed consistent results[47]. However, the true function of the Musashi-1 protein and its relation to GBMSCs still remains elusive.

## CD15

CD15 was recently proposed as the novel putative marker of the stem-like cells derived from brain tumors. However, this has not yet been validated. CD15 is the trisaccharide 3-fucosyl-N-acetyllactosamine (FAL), also known as stage-specific embryonic antigen 1 or LeX antigen, and is highly expressed in many types of pluripotent stem cells, including pluripotent embryonic stem cells, in germinal zones of the developing CNS, in some astrocytes in the adult CNS, and within adult neurogenic zones. NSCs in adult brains can be recognized by the surface marker CD15[48]. A recent study proposed that, in addition to the traditionally recognized NSCs that reside in the SVZ, CD133+ cells (excluding CD24+ cells), in the ependyma may represent another population of NSCs[49]. Glioma-derived neural spheres showed CD15 expression. It was also recognized as a marker for tumor-propagating cells in medulloblastoma[50]. The results of the *in vitro* study of tumor stem cells derived from patients' glioma samples demonstrated that CD15 was expressed in both glioma- and ependymoma-derived tumor spheres, and that its expression decreased dramatically in differentiated tumor spheres[51].

## Sox2

Sox2, an HMG box transcription factor, is expressed in multipotent NSCs. Moreover, a prospective clonal analysis proposed that neurospheres, whether isolated from the embryonic CNS or the adult CNS, expressed Sox2, and Sox2 knockdown mice showed significant defects in the neural stem/progenitor cells[52,53]. In humans, the Sox2 expression is conserved, and heterozygous mutations in this gene cause hippocampal defects and forebrain abnormalities[54]. The correct expression of Sox2 at early stages may be required to establish a downstream transcriptional program for differentiation, perhaps by generating a "poised" chromatin structure at loci crucial for subsequent neuronal development[55,56]. Sox2 may participate in the different networks of transcription factors in stem cells vs. differentiating cells. When overexpressed, it silences the endogenous GFAP activity in differentiating neural cells[56,57]. The study of mRNA expression of *SOX2*, *NOTCH1*, *ID1*, *ASCL-1*, *NEUROD1*, *NEUROG1*, *NEUROG2*, and *NRG1* genes in embryonal supratentorial primitive neuroectodermal tumors (PNETs) showed significantly higher levels of *SOX2*, *NOTCH1*, *ID1*, and *ASCL-1* transcripts[58]. Considering the fact that supratentorial PNET predominantly has glial features, it might be interesting to examine these gene expression levels in high-grade gliomas. Growing evidence suggests that Sox2 may be a tumor marker of glial lineages rather than a universal brain tumor stem cell marker. The aberrant coexpressions of Sox2 and other neuronal markers may also mirror a disorganized differentiation pattern that characterizes highly malignant tumors[59]. A comprehensive study of CXCR4 in GBMSCs surprisingly showed widespread positive staining of both Sox2 and Mushashi-1 in aggressive cells with large pleomorphic nuclei. In particular, Sox2 appeared to demonstrate nuclear localization within neoplastic cells in contrast to a predominantly cytoplasmatic expression in histologically normal adjacent tissue, which demonstrates an activation-induced nuclear translocation of this transcription factor within GBMs. Further confirmation of the Sox2+ GBMSCs revealed the coexpression of additional stem-related markers, such as c-kit, SCF, ataxin-1, Oct-4, and Fut4[60].

## CXCR4

Neurons express a variety of chemokine receptors that regulate neuronal signaling and survival, including CXCR4. The CXCR4 signaling pathway is the key regulator of many essential biological processes, such as stem cell motility, differentiation switch, angiogenesis, apoptosis, and lymphocyte homing. CXCR4 and its only known ligand for CXCL12/SDF-1 are constitutively expressed in the developing and adult brain by both neuronal and glial cells, and are implicated in neurogenesis and connectivity[61,62,63,64]. SDF-1μ possibly regulates the survival of both progenitors and mature neurons. On the other hand,

CXCR4 (a known mediator of proliferation, invasion, and dissemination) is overexpressed in primary glioblastoma progenitor cells vs. corresponding differentiated tumor cells[65,66]. The coexpression of Sox2 was observed in a significant number of CXCR4+ cells[60]. Furthermore, Stevenson et al. established a robust, but significant, connection between CXCR4 expression and the radiographic evidence of increased disease dissemination as observed on T2-weighted MRI, a routinely employed and readily available imaging modality used in the management of patients with brain tumors[67].

It seems that chemokine receptors largely contribute to neuronal survival by maintaining them in a quiescent state[68]. The inappropriate activation of the CDK/Rb/E2F-1 pathway, which possibly results in an abortive attempt of the cell to re-enter the cell cycle, has been implicated as a major mechanism of neuronal apoptosis. E2F-1, one of the major Rb targets, simultaneously activates apoptotic genes *Apaf-1*, *P53*, and *Bax*, as well as the genes required for entry into the S phase[68,69,70]. CXCR4 and SDF-1μ modulate the expression levels and phosphorylation of both Rb and E2F-1, whereas SDF-1μ shows a neuroprotective effect[71]. Thus, the overexpression of the CXCR4 on aggressive GBMSCs may be involved in their survival, activating several antiapoptotic pathways: PTEN/PI3K/AKT, CREB, and NFμB, among others[68]. The biological relevance of CXCR4–ligand interaction was further demonstrated by showing the significant production of CXCL12 protein within human GBMs, particularly by the neovascular endothelium, which indicates the presence of an abundant *in vivo* source of the CXCL12 ligand for activation of CXCR4 signaling in GBM progenitor cells[60,66,67,72,73]. Taken together, these results implicate an important role for the CXCR4 signaling mechanism in GBMSC biology and point to the therapeutic potential of targeting this pathway[74]. It would be of particular interest to investigate the cooperation of CXCR4 signaling and antiapoptotic and stem cell regulatory pathways in an attempt to determine novel molecular targets specific for GBMSCs.

## CD44

CD44 is one of the most prominent stem cell markers and a well-known cancer stem cell marker[75,76,77]. It represents the polymorphic family of cell adhesion molecules that seem to play the key role in the mechanism of tumor invasion and metastasis. The brain tumor cell expression of CD44 may be one of the factors conditioning the highly disparate ability to penetrate the surrounding tissue. Immunohistochemistry of the CD44 standard form (CD44s) and the variant isoforms containing the domain encoded by the exon 3 variant (CD44v3) showed a CD44s+/CD44v− expression profile in GBMs[78]. The immunoelectron misroscope study revealed CD44 expression at the invasive front of the tumor[79]. The overexpression of CD44 could be relevant in determining the highly invasive behavior of gliomas, although it does not behave as an independent prognostic factor for survival[80]. CD44 is up-regulated in GBMs, and its depletion blocks GBM growth and sensitizes GBM cells to cytotoxic drugs *in vivo*. Consistent with this observation, CD44 antagonists potently inhibit the growth of glioma in preclinical mouse models. It seems that CD44 functions upstream of the mammalian Hippo signaling pathway and promotes tumor cell resistance to reactive oxygen species–induced and cytotoxic agent–induced stress by attenuating the activation of the Hippo signaling pathway[81]. The microarray analyses of clinical specimens from glioblastoma patients were used to evaluate potential tumor markers. The expression of the CD44 was elevated in more severe tumor types and was higher in tumor cores than in peripheral regions. On the other hand, lower levels of CD44 expression surprisingly correlated with lower survival[82]. Nevertheless, the CD44 antigen is a promising candidate for further development as a prognostic and therapeutic tool.

## Side Population (SP) Cells

SP cells could be defined as a small subpopulation of cells with an ability to actively discard certain molecules taken up by cells (such as the dye Hoechst 33342) mediated mainly by the overexpression of

ATP-binding cassette (ABC) transporters[83,84,85]. Therefore, the SP cells may be efficiently isolated by flow-cytometry sorting. SP method application has drawn special attention to the field of cancer stem cell research and many studies utilize this method to enrich candidate cancer stem cells from various solid tumors[83,84,86,87,88]. Work by Hirschmann-Jax et al. showed that the SP of neuroblastoma cells not only had the characteristics of tumor stem cells (multipotentiality and self-renewal), but were also more resistant to the effects of drugs such as mitoxantrone, suggesting that these cells may contribute to the overall drug resistance phenotype of relapsed or resistant cancers[89]. The SP cells in a transgenic mouse model of glioma, S100β-verbB;Trp53, showed enhanced tumor-initiating capacity, self-renewal, and multipotentiality compared with non-SP cells from the same tumors. Furthermore, gene expression analysis identified 45 candidate genes that were differentially expressed in glioma stem cells. Analyses of xenografted human GBM cell lines and primary human glioma tissues showed that *S100A4* and *S100A6* were expressed exclusively in a small subset of cancer cells and their abundance positively correlated to tumor grade[90]. Recently, Fukaya et al. isolated cancer stem-like cells from human glioma cell lines utilizing the SP technique. Flow cytometry analysis revealed that SK-MG-1, a human GBM cell line, contained the largest number of SP cells among the five analyzed glioma cell lines. The SP cells had self-renewal ability and were capable of forming spheres in a neurosphere culture medium containing EGF and EGF2. Spheres derived from the SP cells differentiated into three different lineage cells: neurons, astrocytes, and oligodendrocytes. The SP cells expressed nestin and showed tumor-forming ability in xenograft assays[91]. Study of the migration potential of an SP fraction in eight human glioma cell lines and in a sample of primary GBM cells showed that the migration of SP cells was lower than that of non-SP cells. Furthermore, there was a distinct SP in GBM cells selected for slow migration, but not in their fast counterparts. Authors proposed that cells underlying the initiation and recurrence of gliomas are able to migrate, albeit less so than other glioma cells in correlation with the clinical observation that most GBMs recur in close proximity to the site of the original tumor[92]. These results indicate that SP cells isolated from GBMs possess the properties of cancer stem cells, including their self-renewal ability, multilineage differentiation, and tumorigenicity. However, the presence and significance of SP cells in GBMs is still being debated. Recently, Broadley et al. showed that sphere formation in GBM cell lines and primary GBM cells could enrich for a stem-like phenotype of increased self-renewal gene expression *in vitro* and increased tumor initiation *in vivo*, but the SP was absent from all sphere cultures. Direct isolation of the SP from the GBM lines did not result in enrichment for stem-like activity *in vitro*, and tumor-initiating activity was lower in sorted SP compared to non-SP and parental cells. Transient exposure to doxorubicin enhanced both the neurosphere forming and the SP cell frequency. Furthermore, doxorubicin treatment altered the cytometric profile and showed some controversial results regarding the SP cells[93]. These data demonstrate that the GBM SP may not necessarily contribute to enrichment of GBMSCs. Future studies may refine the utility of cancer stem cell surface markers and other techniques for the study of brain tumor stem cells through the identification of improved markers and/or additional new methodologies.

## GENETIC AND EPIGENETIC ALTERATIONS IN GBMSCs

Cancer arises from a series of genetic and/or epigenetic disruptions that occur in a few, or maybe even a single, founder cell(s), which in time eventually acquires unlimited and uncontrolled proliferative potential. Understanding the genetic basis for cancer development is inseparably coupled with revealing the genes and signaling pathways involved in tumor stem cell biology. GBMSC behavior is constantly affected by external signals mediated by ligand-receptor systems that activate complex intrinsic molecular machinery employed in self-preservation. Numerous aberrantly expressed genes and signaling pathways have been identified as important in GBM biology, such as *EGFR*, *PTEN*, *INK4a/ARF*, *NF1*, *PDGFRA/IDH1*, *P53*, *IDH1*, *RB1*, and *ERBB2*[1,2,3,94,95,96,97,98]; as well as PTEN/AKT/PI3K, Sonic Hedgehog/GLI, Notch, P53/MDM2/MDM4/p14$^{ARF}$, and p16$^{INK4a}$/CDK4/RB1 pathways[3,94]. The study of signaling pathways critical to normal neural stem and progenitor cells helped to increase our understanding of the mechanisms that drive cancer stem cell–associated tumorigenesis and tumor progression. Experimental data mostly support two possibilities for GBMSCs of origin, which are not mutually exclusive. First, NSCs or early progenitors may be the transformed ones, showing variable differentiation of their progeny during tumor development. Second, more differentiated glia could be transformed by genetic events that lead to a loss of differentiation maintenance[95]. Despite the plethora of genetic alterations likely entailed, there are a discreet number of genetic events and signaling pathways that have been proven to be central to GBMSC initiation and homeostasis.

## Receptor Tyrosine Kinases and Downstream Pathways

The receptor tyrosine kinase (RTK) family mediates the effects of multiple oncogenic growth factor pathways, including EGFR and PDGFR, as one of the best characterized in gliomas. The binding of specific ligands, and the subsequent dimerization of RTKs, activates an intricately complex network of intracellular cascades modulated by G protein–coupled receptors and second messengers, which converge at multiple sites. The RTKs and downstream signaling pathways are the most studied in GBMs[94,95,96,97]. In tumor stem cells, any of the downstream pathways could be dysregulated through several mechanisms. The primary GBM subtype acutely presents itself as a high-grade disease that typically harbors mutations in *EGFR*, *PTEN*, and *INK4a/ARF* (also known as *CDKN2A*), and the secondary GBM subtype evolves from the slow progression of a low-grade disease that classically possesses *PDGF* and *P53* events. Increased EGFR signaling may be the result of *EGFR* gene amplification or the overexpression of one of the constitutively active variants, most frequently EGFRvIII[1,2,3,95,96]. The coexpression of EGF and EGFR in GBMs leads to a potential autocrine loop.

Bachoo et al. demonstrated that combined loss of p16INK4a and p19ARF may be sufficient for astrocyte dedifferentiation in response to EGFR activation. Moreover, transduction of *INK4a/ARF*$^{-/-}$ in stem cells as well as astrocytes (with constitutively active EGFRvIII) induced a common high-grade glioma phenotype[94]. Similarly, a recent publication showed that the constitutive EGFRvIII activity combined with PTEN loss is sufficient to transform murine neural precursors into high-grade gliomas[97]. EGFRvIII-engineered *PTEN*-/- neural stem/progenitor cells formed highly mitotic tumors with nuclear pleomorphism, necrotic areas, and glioblastoma markers. The transformed cells showed increased cell proliferation, centrosome amplification, colony formation in soft agar, self-renewal, expression of the stem cell marker CD133, and resistance to oxidative stress and ionizing radiation. The ERK and PI3K/AKT pathways were activated. The checkpoint kinase 1 (Chk1), the DNA damage regulator, was phosphorylated at S280 by AKT, suppressing Chk1 phosphorylation at S345 in response to ionizing irradiation[97]. In the recent study, GBMSC lines established from three brain tumor patients were examined for the dependence on various growth factors. The data obtained indicated that only EGF promoted sphere formation and enhanced the self-renewal capacities of GBMSCs, including the CD133+ subpopulations among the growth factors (EGF, FGF-2, PDGF, VEGF, and HGF were tested). The tyrosine kinase inhibitors gefitinib (ZD1839) and AG1478 efficiently inhibited EGF-dependent sphere formation, while gefitinib significantly decreased the CD133 populations and also induced their apoptosis[98]. Telomerase reverse transcriptase (TERT), the catalytic subunit of the enzyme telomerase, is robustly expressed in GBMs. Beck et al. reported a telomerase activity-independent function of TERT that might induce cancer stemness in glioma cells. Overexpression of TERT712 in U87MG cells promoted cell self-renewal *in vitro*, and significantly induced EGFR expression and formation of GBMs. In patients with GBM, TERT expression showed a high correlation with EGFR expression and was closely linked to the stemness gene signature. Induction of differentiation and TERT knockdown in glioma stem cells led to a marked reduction in EGFR expression, cancer stemness, and anticancer drug resistance[99]. However, although EGFR is highly expressed in many GBMs, anti-EGFR therapies have been unsuccessful as treatment. More studies are needed to examine the EGFR activation in GBMSCs compared to other GBM cells to determine if patient-specific GBMSCs are amenable to anti-EGFR therapy preclinically. In contrast to previous reports, a recent study showed that GBMSCs proliferated in the absence of exogenous mitogenic stimulation (including EGF and FGF-2) and gave rise to multipotent GBM spheres that were capable of self-renewal. EGF and FGF-2 only enhanced GBMSC survival, proliferation, and subsequent sphere size. Implantation of as few as 10 exogenous mitogen-independent GBMSCs led to the formation of highly invasive intracranial tumors, which closely resembled human GBMs, in immunocompromised mice. These results suggest that exogenous mitogen independence and/or partial dependence, mediated through EGFR signaling, is one of the important characteristics that may distinguish GBMSCs from neural stem cells[100]. Howard et al. hypothesized that EGFR activation in GBMSCs varies between patients, presenting as an important determinant of responsiveness to anti-EGFR therapy. Only EGFR+/PTEN+ GBMSC clones were shown to be susceptible to anti-EGFR therapy *in vitro*[101]. Furthermore, Mazzoleni et al. showed that multiple distinct GBMSC subpopulations could be purified from the specimens of patients with GBM and from cancer stem cell lines based on the expression of EGFR and of other putative cancer stem cell markers. All these subpopulations were molecularly and functionally distinct and tumorigenic, but EGFR-expressing tumor-initiating cells displayed the most malignant functional and molecular phenotype. Accordingly, modulation of EGFR expression by gain-of-function and loss-of-function strategies in GBMSC lines enhanced and reduced their tumorigenic ability. These findings open up the possibility of new therapeutically relevant scenarios, as the presence of functionally heterogeneous $EGFR^{pos}$ and $EGFR^{neg}$ GBMSCs subpopulations within the same tumor might affect clinical response to treatment[102].

RTK signaling is transduced and further amplified through the downstream prosurvival AKT/PI3K pathway. On the other hand, the loss of the *PTEN* function, whether via gene mutations/deletions or promoter hypermethylation, results in an aberrant AKT overexpression, promoting the survival, proliferation, invasion, and secretion of proangiogenic factors[95,103,104]. Animal models have also demonstrated that PTEN loss is functionally equivalent to AKT activation when combined with activated Ras[103,104]. Somatic mutations in *PTEN* occur frequently as late events in sporadic brain tumors. Models based on deletion of this gene in the mouse brain have provided insight into the normal functions of this gene in the neural stem pool, as well as in the initiation and progression of gliomas. The simultaneous CNS-specific deletion of *P53* and *PTEN* in the mouse CNS resulted in a penetrant acute-onset high-grade malignant glioma phenotype with a notable clinical, pathological, and molecular resemblance to GBM in humans. Furthermore, integrated transcriptomic profiling and functional studies of murine NSCs further established that the dual inactivation of *P53* and *PTEN* exclusively promotes an undifferentiated state with high renewal potential and increases Myc protein levels and its associated signature. These data, in addition to the confirmatory transcriptomic/promoter studies in human primary GBM, stress Myc as an important target for cooperative actions of *P53* and *PTEN* in the regulation of normal and malignant stem/progenitor cell differentiation, self-renewal, and tumorigenic potential[105]. PI3K-activating mutations are found in a surprising number of patients with glioblastoma, as indicated by the phosphorylation of key signaling proteins in the PI3K pathway[106].

Regardless of ligand or receptor status, almost 100% of GBMs exhibit the activation of Ras[107,108]. Ras-GTP is downstream of the growth factor receptors at a major signal transduction crossroad, translating extrinsic messages into the Raf-MAPKK-ERK pathway or into either the PI3K-PKB or the PI3K-Rac-Rho pathway, directly influencing cell survival and migration, allowing it to influence most tumor cell behaviors. There is some evidence that the cooperation of Ras/AKT signaling may be essential for GBMSC maintenance. Combined activation of both Ras and AKT induced high-grade gliomas with the histological features of human GBMs. Significantly, these tumors raised almost exclusively after gene transfer to neural progenitors, but not after transfer to differentiated astrocytes[109]. p-MAPK and the activated members of the AKT pathway were proposed as the markers of outcome in GBM patients. Furthermore, the elevated expression of p-MAPK was directly associated with the increased radiation resistance[110]. Due to the association of the AKT pathway activity with a wide range of tumorigenic properties, it is possible that GBMSCs may exhibit some level of dependence on the AKT pathway. A recent study demonstrated that GBMSCs are more dependent on AKT signaling than matched nonstem glioma cells. Pharmacologic inhibitors of AKT attenuated glioma cancer stem neurosphere formation, induced apoptosis in a significant number of GBMSCs, and substantially delayed the intracranial tumor formation[111]. The local delivery of an AKT small-molecule inhibitor was effective against the experimental intracranial glioma, with no observed resistance to GBM cells grown in stem cell conditions[112]. These data suggest that AKT inhibition may specifically target the GBMSC population to reduce tumor malignancy.

## Sonic Hedgehog/GLI Pathway

Sonic Hedgehog (SHH) is one of the key regulators of neural progenitors in development, and have also been found to be altered or overexpressed in GBMSCs[113,114]. The binding of Hedgehog ligands to their receptors activates transducers termed GLIs (named for their discovery in gliomas), which then translocate into the nucleus to activate or repress downstream targets. It was demonstrated that SHH-GLI signaling may regulate the expression of stemness-related genes and the self-renewal of CD133+ GBMSCs. SHH-GLI signaling was required for sustained glioma growth and survival[114]. A recent paper proposed that GLI also requires NANOG activity, forming a positive loop, which is negatively controlled by P53 and vice versa[115]. Furthermore, the SHH-GLI pathway displayed additive and synergistic effects with temozolomide, the current chemotherapeutic agent of choice, which showed no effects on GBMSCs. Moreover, the treatment of GBMSCs with the Hedgehog inhibitor cyclopamine or transduction with GLI-RNA interference inhibited proliferation and self-renewal and increasing apoptosis[114]. Bar et al. showed that cyclopamine treatment depletes GBMSCs, as viable cells after treatment failed to propagate tumors *in vivo*. Cyclopamine treatment also improved the effects of radiation on GBMSC survival[116]. Together, these data suggest that the Hedgehog pathway pharmacologic inhibitors may improve traditional therapy efficiency against gliomas.

## Notch Pathway

Physiologically, Notch signaling promotes the proliferation of normal NSCs, suppressing their differentiation[117,118]. Notch was first linked to GBMSC homeostasis due to the observation that Notch signaling increases expression of the nestin in gliomas. Notch expression in a K-Ras–induced mouse glioblastoma model resulted in the glioma formation in the NSC-rich subependymal zone[119]. Arsenic trioxide ($As_2O_3$) showed a remarkable inhibitory effect on GBMSCs in human glioma cell lines (U87MG, U251MG, and U373MG) *in vivo* and *in vitro*, suggesting that the deregulation of Notch activation may impair GBMSC homeostasis[120]. Wang et al. showed that the expression of the constitutively active intracellular domains of Notch1 or Notch2 protect glioma stem cells against radiation, while inhibition of the Notch pathway with γ-secretase inhibitors (GSIs) rendered the glioma stem cells more sensitive to radiation at clinically relevant doses. Surprisingly, Notch inhibition with GSIs did not alter the DNA damage response after radiation, but rather reduced AKT activity and Mcl-1 levels in glioma stem cells. Notch inhibition did not affect nonstem glioma cells[121]. These data suggest that inhibition of Notch signaling holds promise to improve the efficiency of current radiotherapy targeting the GBMSC population. Notch signaling seems to be linked to the contact of GBMSCs with the vascular niche. A recent study proposed that the perivascular niche may activate Notch signaling, which may drive tumor stem-like characteristics in gliomas[122,123]. Another recent study showed that NO from the perivascular niche could activate Notch in PDGF-induced gliomas. eNOS expression was highly elevated in the tumor vascular endothelium adjacent to perivascular glioma cells expressing nestin, Notch, and the NO receptor sGC. *In vitro* studies suggested that the NO/cGMP/PKG pathway could regulate Notch signaling in PDGF-induced gliomas and induce the side population phenotype in primary glioma cell cultures. NO also increased the neurosphere-forming capacity and enhanced their tumorigenic capacity *in vivo*, while loss of Notch signaling due to NO absence prolonged survival of mice[122]. The NO/cGMP/PKG pathway's promotion of the stem cell–like character in the tumor perivascular niche may identify therapeutic targets for a PDGF/PDGFR-related subset of gliomas. Notch inhibition in GBM explants resulted in decreased proliferation and self-renewal of tumor stem cells, but was also associated with a decrease in endothelial cells. Again, combination treatment with Notch blockade and radiation resulted in a substantial decrease in proliferation and self-renewal in tumor explants[123]. The Notch pathway seems to play a critical role in linking angiogenesis, response to hypoxia, and cancer stem cell self-renewal presenting as a promising therapeutic target.

## Epigenetic Mechanisms

Epigenetic mechanisms are extremely powerful regulators of tumor stem cell viability, opening a promising new area of research. Genome-wide methylation pattern studies revealed that global hypomethylation occurred at a high frequency (~80%) in primary GBM. The most severe globally hypomethylated cases were also the most progressive ones and showed the association with demethylation, and therefore activation, of the putative onogene *MAGEA1*. The demethylation of repetitive DNA sequences, such as the tandem repeat satellite 2 located at the juxtacentromeric regions of chromosomes 1, 9, and 16, and the D4Z4 repeat at the subtelomeric regions of chromosomes Chr4q35 and Chr10q26, contributed to the DNA hypomethylation phenotype in GBMs[124]. The molecular and functional consequences of global hypomethylation in GBMs as well as in GBMSCs are not yet elucidated.

On the other hand, the locus-specific CpG island hypermethylation of the promoters of genes related to DNA repair, apoptosis, invasion, angiogenesis, and drug resistance is frequent in GBMSCs. Promoter hypermethylation regulates TGF-β signaling in GBM through the induction of PDGF-B, among others[125,126]. Bone morphogenic proteins (BMPs) are a family of growth factors that belong to the TGF-β superfamily. BMPs bind to their cell-surface receptor kinases (BMPRs) and activate SMADs phosphorylation[125]. The role of BMPs in neurogenesis is very complicated, promoting proliferation, apoptosis, or the differentiation of neural stem and/or progenitor cells under certain conditions[127]. A differentiation-stimulating response to BMPs seems to be preserved in GBMSCs. A recent study revealed that this differentiation pathway in GBMSCs is commonly inhibited by the epigenetic silencing of the *BMPR1B*, maintaining them in a stem-like state. This silencing is dependent on the histone methyltransferase enhancer of zeste 2 and DNA methylation, and may be reversed by treatment with a DNA demethylating agent. Furthermore, the forced expression of *BMPR1B* could restore the differentiation potential of these cells, thus decreasing tumorigenicity[128].

The polycomb family of genes represents epigenetic effectors involved in CNS development and cancer progression. Physiologically, DNA methylation and polycomb-mediated histone H3 K27 trimethylation largely contribute to repressing pluripotency and define the developmental potential of progenitor cells in CNS[129]. In GBM, the aberrant expresson of polycomb genes (especially *Bmi-1* and *EZH2*) inhibits differentiation and activates transformation- and invasion-related genes and sustains GBMSC self-renewal[130,131,132]. Study of the malignant potential of NSCs from wild-type and *Bmi-1* knockout mice showed that Bmi1 is required for transforming both differentiated astrocytes and NSCs[130]. It seems that *Bmi1* expression represses alternate tumor-suppressor pathways that attempt to compensate for *INK4a/ARF/P53* deletion and PI3K/AKT pathway hyperactivity. Inhibition of EZH2, the main component of the polycomb-repressive complex 2, impaired GBMSC growth as well. Specific shRNA-mediated down-regulation of EZH2 in combination with chromatin immunoprecipitation experiments revealed that c-Myc could be a direct target of EZH2 in GBMSCs. This direct transcriptional regulation of c-Myc by EZH2 may constitute a novel mechanism underlying GBM cancer stem cell maintenance and suggest that EZH2 may be a valuable new therapeutic target for GBM management[132].

miRNAs are powerful intracellular regulators that can silence target genes through post-transcriptional and post-translational mechanisms. miRNAs are emerging as important regulators of cellular differentiation and proliferation, and have been implicated in the etiology of a variety of cancers, including GBMs. The study of the role of miRNAs in GBMSCs revealed that miR-124 and miR-137 were underexpressed in GBM and anaplastic astrocytoma in comparison with the normal brain. The targeted delivery of these two miRNAs inhibited proliferation due to the differentiation of GBMSCs[133]. There are putative binding sites of NF-κB and c-Myc in the promoters of several up-regulated miRNAs in GBM, indicating these transcription factors may also be involved in the regulation of miRNA expression, thus affecting the activity of the AKT pathway in tumorigenesis[134]. Comparing the miRNA profiles of GBMSCs and nonstem GBM cell populations showed the down-regulation of several miRNAs in the GBMSCs, including miR-451, miR-486, and miR-425. Transfection with the miR-451, miR-486, and miR-425 inhibited the formation of the neurosphere, and the transfection with the mature miR-451 dispersed neurospheres and inhibited GBM cell growth. Furthermore, the transfection of miR-451 combined with imatinib mesylate treatment had a synergistic effect and resulted in the dispersal of GBM neurospheres. The promoter region of miR-451 was identified as the target site for SMAD3 and 4, and the transfection of SMAD in GBM cells inhibited their growth, suggesting that SMAD may drive GBMSCs to differentiate through the up-regulation of miR-451, reducing their tumorigenicity[135]. Kefas et al. showed that expression of miR-7 is down-regulated in GBMs, likely through a processing defect. The transfection with miR-7 inhibited expression of both the EGFR and of upstream drivers of the AKT pathway[136]. Notch is regulated by a number of miRNAs, including miR-1, miR-34, miR-146, miR-199, and the Let-7 family of miRNAs, while aberrant down-regulation of these miRNAs is often implicated in the development of various cancers, including gliomas[137,138]. Therefore, these miRNAs are often selected as candidates or novel anticancer therapeutics. Recently, Kefas et al. demonstrated that in both glioma stem cells and nonstem tumor cells, Notch knockdown with small interfering RNAs could stimulate up-regulation of miR-326. In addition, the transfection of both glioma stem and nonstem cells with miR-326 showed a decline in Notch signaling and subsequent reduction in the viability, proliferation, and invasiveness *in vitro*, as well as the tumorigenicity *in vivo;* indicating a feedback loop, the “Notch/ miR-326 axis”[139]. Similarly, it was demonstrated that miR-34a suppresses brain tumor growth by targeting both c-Met and Notch[140]. The identification of additional miRNAs and their specific target genes that regulate GBMSC homeostasis may provide new potential drugs for therapy.

The characterization and molecular targeting of the key regulator molecules specific for the GBMSCs could lead to new therapeutic strategies in the future. Targeting GBMSCs will likely have to rely on genomic or proteomic characteristics that differ from other glioma tumor cells. The GBM subtypes should also be kept in mind, as their GBMSCs constitute distinct genetic disease entities and likely involve different genetic alterations and signaling pathways. There is promising and mounting evidence that future GBM therapies, guided by patient-specific epigenetic profiling, might be applied more rationally. Due to the reversible nature and the key role in the gene expression pattern, epigenetic alterations, especially the changes in histone acetylation, have already been exploited for therapeutic drug targeting in several clinical trials. The powerful next-generation sequencing methodology will accelerate GBMSC epigenome profiling, providing new predictive markers and novel therapeutic strategies. On the other hand, mounting evidence suggests that specific miRNA expression could inhibit various malignancy end points in glioma stem cells. It seems that miRNAs hold promise as potential potent therapeutic agents that may act by targeting multiple oncogenic pathways in brain tumors and by inducing the differentiation of cancer stem cells. However, most of the studies have not clearly delineated any differences between glioma cells and glioma stem cells. Therefore, further research into the role of miRNAs and GBMSC signaling pathways may better support this innovative therapeutic approach.

## GBMSCs AND THE IMMUNOSUPPRESSION MECHANISMS

Many novel therapies for cancer patients are designed to target the immune system and boost the antitumor response, such as monoclonal and chimeric antibodies, interferons and other cytokines, anticancer vaccines, and chemotherapeutics that promote specific antitumor immunity[141,142,143,144, 145,146,147]. Therapy with dendritic cells and immunogene vaccines showed very promising results in preclinical glioma studies[147,148,149,150]. However, these could not be translated into effective clinical therapies despite years of active research. Local and systemic immunosuppression created by GBM may be the key obstacle for an adequate therapeutic antitumor immune response. The well-documented disruption of the immune system of GBM patients is manifested as a peripheral low leukocyte count (predominantly induced by the lymphocyte number decrease), impaired blastogenic responses by the peripheral blood mononuclear cells (PBMNCs), down-regulated phagocytic and oxidative burst ability, disrupted antigen recognition, and reduced type-IV hypersensitive reaction to recall the antigens[151,152,153,154,155,156].

To date, knowledge of the complex coordination of antitumor immune responses within the brain remains limited. The precondition for the development of effective immunotherapy strategies for GBM is an adequate understanding of the unique microenvironment in the CNS. The hypothesis that the brain is "immunologically privileged" is being replaced with a standpoint that it is actually a specialized immune site under tight regulatory control[157,158,159,160]. Therefore, it represents a unique tumor microenvironment. GBMSCs are considered to be responsible for tumor maintenance and recurrence, but their role in creating local and systemic immunosuppression has not yet been completely elucidated.

Brain tumor-infiltrating lymphocytes (TILs) provided strong evidence that the immune system is naturally involved in the immunosurveillance of brain tumors. In addition, the presence of tumor-associated macrophages (TAMs) within the GBM microenvironment has been associated with enhanced tumor progression and poor prognosis[161]. It is accepted that TAMs have multiple roles promoting GMB growth and spread, angiogenesis, and immunosuppression[162,163,164,165]. These paradoxical roles of macrophages in numerous cancers find an explanation in their functional plasticity, which may result in the polarized expression of either pro- or antitumoral functions due to microenvironmental signals to which macrophages are exposed, which selectively tune their functions within a large functional spectrum[162,163]. TAMs have been described in some studies as relatively immature cells, characterized by low expression of the differentiation-associated macrophage antigens, carboxypeptidase M and CD51, high constitutive expression of interleukin (IL)-1 and IL-6, and low expression of TNF-α[166,167]. It seems that at least four different microenvironments — sites of initial tumor cell invasion, perivascular sites, stromal regions, and hypoxic/necrotic areas — may "educate" macrophages to carry out specific functions in support of tumor cell requirements and activities in those different areas. Therefore, TAMs and resident microglia could be "educated" towards several tumor-promoting phenotypes with various functions by key regulators of the GBM homeostasis[160,164]. Specialized subpopulations of TAMs may represent important new therapeutic targets[168]. In this review, we focus on the mechanisms underlying local and systemic immunosuppression in GBM patients in an attempt to understand some of the obstacles for the development effective immunotherapeutic strategies, particularly those targeting GBMSCs.

It has been well established that GBM cells utilize "classical" tumor-immune escape mechanisms, such as the down-regulation of the costimulatory molecules, producing immunosuppressive cytokines TGF-β, PGE2, IL-10, gangliosides, as well as defective dendritic cell function[169,170,171,172]. Another mechanism is killing effector T cells via apoptosis (utilizing the immunosuppressive protein B7-H1 in direct GBM stem/T-cell contact, or by the secretion of Galectin-3)[171,173,174,175,176,177,178,179]. All denominated processes are influenced by the intrinsic properties of GBM cells, particularly GBMSCs, as well as microenvironmental factors.

## GBM-Associated Immunosuppressive Immune Cells

A large number of studies suggest that certain types of tumor microenvironmental immune cells actively affect tumor development and progression. Inflammatory cells, primarily macrophages/microglia and regulatory T cells, may affect these processes via their ability to express a large variety of factors, including immunoregulatory cytokines. These cytokines secreted by inflammatory cells, tumor cells, and stroma cells together establish a network of factors that significantly affect GBM evolution and provide conditions for GBMSC maintenance. This highlights an increasingly important concept in GBM

pathogenesis: immunosuppression does not result exclusively from glioma-derived factors. While glioblastoma cells express multiple immunosuppressive factors, including TGF-β2, PGE2, IL-10, gangliosides, and B7-H1, the effects of these factors are likely to be local and cannot explain many of the systemic immune defects seen in glioblastoma patients[179,180,181]. A new study links the progression of a lethal type of brain tumor with a reduced expression of more than 600 immune system genes, suggesting how complex the immune response is to the cancer and the resulting difficulty in targeting specific immune system proteins for treatment[182].

## Tregs

T regulatory cells (Tregs) belong to a “self-check" compartment built into the immune system to prevent excessive reactions. Tregs are comprised of a few subpopulations, including the most studied CD4+CD25+FoxP3+ Tregs. Physiologically, in peripheral blood, Tregs represent 5–10% of the CD4+ T-cell population. They induce immune tolerance inhibiting dendritic cell maturation and their antigen-presentation ability, T-cell activation, and proliferation in order to prevent autoimmune responses. The mechanism of T-cell suppression is contact dependent and often mediated by IL-10 and TGF-β[183,184]. Recently identified human CD8+CD25+ lymphocytes are also capable of suppressing allogeneic and autologous T-cell proliferation in a cell contact–dependent manner, mediated by TCR/HLA-E/self peptide interactions[185,186]. A phenotypically distinct CD8+ Treg cell population was identified in the ovarian carcinoma microenvironment. These IL-10+/CCR7+/CD45RO+/CD8+ cells were induced by the plasmacytoid dendritic cells in tumor ascites. They were found to significantly inhibit mature myeloid dendritic cell–mediated tumor-associated antigen-specific cytotoxic T-effector function through IL-10 production[186]. Recent studies have shown that *in vivo* depletion of Tregs mediates the regression of tolerogenic tumors[187,188,189]. The immunotherapy trial employing Ontak (IL-2 fused to diphtheria toxin) to deplete Tregs in ovarian cancer patients additionally showed an increased percentage of IFN-expressing CTL without inducing autoimmune reactions[189].

Several independent studies confirmed that Tregs infiltrate malignant gliomas[174,175,190]. In a mouse glioma model, the reversible specific depletion of Tregs significantly increased survival, indicating the relevance of Tregs in GBMs[175,176]. Grauer et al. demonstrated a time-dependent increase in the Treg number in a syngenic murine glioma GL261 model. The expression of regulatory molecules such as CD25, CTLA-4, GITR, and CXCR4 on intratumoral Tregs also showed up-regulation in a time-dependent manner. Combining Treg depletion with the administration of blocking CTLA-4 monoclonal antibodies further boosted glioma-specific CD4+ and CD8+ effector T cells, as well as antiglioma IgG2a antibody titers, resulting in complete tumor eradication[176]. This study illustrated the significance of the intratumoral accumulation and the level of activation of Tregs as an important immune escape mechanism for GBM, and indicated the importance of controlling tumor-infiltrating Tregs in glioma immunotherapy.

An increased FoxP3+ Tregs to CD4+ T cells ratio correlates with the impairment of CD4+ T-cell proliferation in peripheral blood specimens obtained from patients with GBM[190,191]. However, the data on regulatory cell fractions in the blood and the prognosis of GBM patients are still sometimes contradictory[190,191,192]. Furthermore, studying human Tregs is complex, as human-activated nonregulatory CD4+ T cells transiently express both CD25 and FoxP3. Several studies have demonstrated that the down-regulation of the IL-7 receptor (CD127) on Tregs distinguishes Tregs from other T cells, providing a novel important marker for GBM immunosuppression analyses[193,194,195].

The immunophenotypization of the lymphocyte fractions in ultrasonic aspirated material from human brain tumors detected that CD4+FoxP3+CD25$^{high}$CD127$^{low}$ Tregs accumulate specifically in high-grade brain tumors. Tregs in human glioblastoma tissues expressed significantly higher levels of Treg-activating markers FoxP3 and CTLA-4 compared to the same lymphocyte population in the blood[196]. Tregs were not obtained in normal brain tissue and were very rarely found in low-grade gliomas and oligodendrogliomas[196,197]. The Tregs in the tumor microenvironment strongly suppressed TIL proliferation and cytokine production. Immunohistochemical data showed that brain tumor–infiltrating

Tregs are mainly localized perivascularly, and that a smaller fraction of Tregs infiltrate deep into the tumor, always in close proximity to other lymphocytes. This may allow the Tregs to exert their contact-dependent suppression *in vivo*[176,196]. Moreover, accumulation of Tregs strongly correlates with the WHO grade[176,196,197]. A hypothesized parameter that may be more predictive than solely the level of Tregs in GBM could be the balance between cytotoxic T cells and Tregs, as well as CD8+ regulatory T cells (effectors/suppressors ratio).

One of the key questions is whether the accumulation of Tregs is due to the specific tumor recruitment or to the local expansion in the tumor microenvironment. Animal models have shown that Tregs can proliferate fast and that their turnover *in vivo* is short[193,196]. The study of the proliferative potential of the GBM cells and tumor cells in brain metastases showed a strong and significant decrease of MIB-1 expression, which implies that the capacity of Tregs to proliferate probably declines as they infiltrate the tumor[196]. These data imply that the specific accumulation of Tregs in high-grade brain tumors is not primarily due to their proliferation at the tumor site and support findings that GBM cells, and particularly GBMSCs, secrete a large amount of cytokines that may recruit Tregs. The structural and functional abnormalities in the vascular microenvironment in GBM, causing the loss of blood-brain barrier function, possibly further influence Treg infiltration. In addition, the brain tumor microenvironment possibly favors Treg survival and function[198,199,200]. Recent studies are mostly focused on the relationship between GBMSCs, Tregs, and other TILs. It was demonstrated that GBMSCs inhibited T-cell proliferation and activation, induced regulatory T cells, and triggered T-cell apoptosis, mostly due to the activated form of signal transducer and activator of transcription 3 (STAT3), which will be discussed in detail later.

## Macrophages/Microglia

GBMSCs have been shown to suppress adaptive immunity using complex multilevel mechanisms, but their role in modulating innate immunity is even more enigmatic. Furthermore, a recent study demonstrated the role of microglia in the CNS development[201]. In the CNS, macrophages/microglia constitute the first line of defense against a variety of stressors and are able to perform a broad range of functions, participating in the regulation of innate and adaptive immune responses[178,202]. Upon activation, these cells release reactive oxygen intermediates and proteinases, become capable of phagocytosis, and up-regulate MHC and costimulatory molecules modulating both the CD4-specific and CD8-specific T-cell response[203]. On the other hand, both microglia and macrophages in the CNS are capable of a potent suppression of the local immune system. Many human gliomas exhibit prominent macrophagal/microglial infiltrates, which can account for as much as 30% of the tumor mass[202,204]. Furthermore, the abundance of infiltrates correlate with the grade of malignancy and vascular density in the tumors[205,206,207,208,209].

Several studies have shown that the GBM-infiltrating macrophages/microglia are morphologically and phenotypically distinct from the resting microglia present in the intact brain[210,211]. Histopathologic studies of GBM have consistently shown microglial infiltration throughout the tumor, rather than the areas of necrosis, while the phagocytosis of the tumor cells and debris have not been observed[206,207,212]. It was postulated that the defense functions of macrophages/microglia against GBM are compromised in the tumor microenvironment[206]. Furthermore, recent evidence strongly supports that this particular cell population contributes to the immunosuppressive environment of gliomas and promotes tumor proliferation and progression[172,207,213,214,215,216,217]. It has been demonstrated that the invasive potential of GBM was lower in macrophage/microglia–depleted organotypic brain culture, but the addition of microglia to the microglia-depleted slices restored the invasiveness[211]. GBM "educated" macrophages/microglia were unable to produce proinflammatory cytokines, but released IL-10 upon stimulation, induced effector T-cell anergy, demonstrated impairments in cytotoxicity, and induced Tregs[206,214,218,219,220,221]. These phenomena were reproduced with both the T-cell– (CD40L) and pathogen-derived (TLR5 and TLR8 ligands) stimuli, demonstrating that

GBM affects these cells regardless of the nature of the stimulus. Surprisingly, some of these features are shared, although to a much lesser degree, by nontransformed human astrocytes *in vitro*, suggesting that human astrocytes create a regulatory network that prevents monocytic/microglial activation, an ability that is exacerbated in highly malignant gliomas[214]. Recently, Wu et al. demonstrated that GBMSCs largely contribute to the conversion of peripheral blood monocytes to immunosuppressive macrophages/microglia due to activity of secretory factors such as sCSF, TGF-β1, and MIC-1. The single-cell suspensions from the GBM samples, which contained a low frequency of GBMSCs, failed to significantly induce the change of the monocytic phenotype[217]. The GBMSC-conditioned medium potently polarized macrophages/microglia into the immunosuppressive phenotype, inhibited phagocytosis, induced the secretion of the immunosuppressive cytokines, such as IL-10 and TGFβ1, and enhanced their capacity to inhibit T-cell proliferation[217].

It seems that upon their recruitment into the GBM microenvironment by chemotactic cytokines and other molecules (such as RANTES/CCL5, CCL2, IP10, VEGF), monocytes and macrophages/microglia adopt a characteristic immunosuppressive phenotype that resembles myeloid-derived suppressor cells (MDSCs)[213,215,217]. MDSCs, a heterogeneous population of immature and mature myelomonocytic cells, have been identified in several cancers and correlate with tumor burden. They have multiple passive and active immunosuppressive effects, including impaired antigen presentation, the ability to induce apoptosis in activated T cells, and the ability to stimulate Treg proliferation[222,223,224]. The study of GBM and monocytic/microglial interactions showed that the inhibition of TNF-α was both soluble and cell-contact dependent, whereas the induction of IL-10 appeared to be entirely cell-contact dependent[214]. Normal human monocytes exposed to GBMSCs adopted a myeloid-derived suppressor-like (MDS-like) phenotype, where they maintained CD11b expression and exhibited reduced CD14 expression (in contrast to almost all other CNS pathologies), but increased the expression of various immunosuppressive molecules (including IL-10, TGFβ, and B7-H1), decreased phagocytic ability, and increased the ability to induce apoptosis in activated lymphocytes. Direct contact between monocytes and GBMSCs was necessary for the complete induction of this phenotype. Moreover, the authors found significantly elevated numbers of circulating MDS-like cells in the peripheral blood of GBM patients, suggesting that these cells may contribute to systemic immunosuppression[213].

The source of intratumoral and peripheral MDS-like cells in GBM patients has not been established. In the case of other cancers, it was proposed that soluble, tumor-secreting factors, such as IL-6, IL-10, PGE2, or TGF-β2, may be responsible for the proliferation of these cells[222,223,224]. However, serum levels of GBM-secreting factors known to stimulate MDCSs and MDS-like cells were not significantly higher compared to normal donors[182,213]. To date, few models have been developed for the purpose of explaining how local factors translate into the profound systemic immunosuppression noted in GBM. The confounding effect of chemotherapy, surgery, radiation, and symptomatic therapy (e.g., dexamethasone) on immunity further complicates this problem. Gustafson et al. investigated characteristics of tumor and dexamethasone-dependent alterations in the monocytic phenotype CD14+HLA-DR$^{lo/neg}$. However, CD14+HLA-DR$^{lo/neg}$ cells could be re-created *in vitro* using dexamethasone-free tumor supernatants and dexamethasone could not induce any significant functional differences, although its presence increased the immunophenotype conversion[215]. CD14+HLA-DR$^{lo/neg}$ cells are of particular clinical interest as predictive and prognostic markers of immunosuppression and poor outcome in various nonmalignant pathologies[225,226,227,228,229,230]. Moreover, this particular myeloid phenotype has been identified in several other cancers[223,224,231,232,233]. Schartner et al. have shown that tumor-associated microglia display an impaired capacity to up-regulate MHC class II, even upon stimulation with potent microglial activators[234]. A positive correlation between CD14+HLA-DR$^{lo/neg}$ monocytes and circulating Tregs was observed[215]. Combined, the data suggest that circulating MDS-like cells in GBM patients may arise from tumor-recruited monocytes and/or macrophages that have undergone an immunosuppressive “education” inside the tumor by GBMSCs. Subsequently, the MDS-like cells released into the circulation may inhibit the adaptive immune response by preventing dendritic cell maturation and multilevel inhibition of T-cell responses. Finally, increased Treg levels might occur in response to stimulation of naive T cells by circulating MDSCs and MDS-like cells.

The molecular mechanisms that mediate the differentiation of the immunosuppressive MDS-like phenotype have not been fully established. However, it was shown that the tolerance induced in monocytes/microglia by GBMSCs is not associated with the interference with the signaling cascades associated with pattern recognition receptors or CD40-induced activation[178,214]. Rather, these tumor cells appear to up-regulate pathways that antagonize positive signaling pathways, including but not limited to STAT3 and STAT5[214].

## STAT3 Regulation of GBMSCs and Immunosuppression

The **s**ignal transducer and activator of transcription 3 (STAT3) is a transcription factor that mediates the expression of a variety of genes in response to cytokines, growth factors, hypoxia, and other exogenous stimuli, and thus plays a key role in many cellular processes such as cell growth, development, apoptosis, regeneration, immunomodulation, and cancerogenesis. Unlike all other members of the STAT gene family, ablation of *STAT3* leads to embryonic lethality. These findings suggest that STAT3 might be more generally deployed than its relatives and has led to the suggestion that it might represent a primordial STAT protein. Recent data, especially from the analysis of conditional loss of the STAT3 protein in adult tissues, confirm that STAT3 participates in a wide variety of physiological processes and even directs seemingly contradictory responses[235]. STAT3 is one of the key regulators of immunosuppression by influencing macrophage activation and dendritic cell function, reducing the cytotoxicity of the NK cells and neutrophils, and limiting the inflammatory response[236].

Considering the variations in the expression of the functional markers of Tregs, it seems that this cell population adapts to the tumor microenvironment, possibly due to the STAT3 activation. On the other hand, STAT3 is a well-known regulator of GBM pathogenesis. The STAT3 protein has been shown to regulate the fundamental components of tumorigenesis and maintenance by preventing apoptosis (by increasing survivin levels, and BCLXL and MCL gene expression), enhancing proliferation (by increasing c-Myc and cyclin D1/D2 expression), and stimulating neovascularization (by increasing VEGF and HIF-1α expression) and invasion (by increasing MMP-2 and MMP-9 expression)[237,238,239,240]. It is overexpressed in most aggressive cancers, including glioblastomas. Recently, it has been shown that STAT3 regulates the growth and self-renewal of GBMSCs and, therefore, it has been proposed as a novel promising molecular target for GBMSC targeted therapy[192,241,242].

STAT3 inhibition in GBMSC cultures (isolated and purified from the patients' tumor tissue) with small-molecule inhibitors (STA-21 and S3I-201) showed a permanent loss of the stem-like characteristics of GBMSCs. After several days of treatment, the cell growth in STAT3-inhibited cultures was minimal compared to the growth in the control cultures. Moreover, the STAT-inhibited cultures showed a rapid down-regulation of the proteins involved in stem cell homeostasis. Strikingly, a single, acute treatment with STAT3 inhibitors was effective *in vitro*[242]. It was proposed that the blockade of this pathway deprives the GBMSCs' ability to maintain and promote tumor growth, inducing the possibility of their differentiation[241]. Furthermore, recent studies have revealed that the activated (phosphorylated) form of STAT3 may be the key mediator in the GBM immunosuppression highly involving Tregs. The levels of phosphorylated STAT3 (p-STAT3) expression significantly correlated with a poor prognosis in patients with anaplastic astrocytomas and with the degree of immune infiltration[240]. Animal experiments have shown that the ablation of STAT3 in the hematopoietic system was accompanied by a reduction in the number of tumor-infiltrating Tregs[243]. STAT3 also proved to be required for both TGF-β and IL-10 production by CD4+ T cells, factors necessary for the generation of tumor-associated Tregs[244]. Furthermore, IL-2 regulates FoxP3 expression in human Tregs, possibly by STAT3 binding on the first intron of the FoxP3 gene[245]. The CD133+ cancer-initiating single colonies from GBM patients, with a strong ability to inhibit T-cell proliferation and activation, which induce Tregs and trigger T-cell apoptosis, showed a constitutive STAT3 expression[241,244]. The STAT3 blockade (by silencing with miRNAs or by small molecular inhibitors, such as WP1066) was able to restore the T-cell proliferation and the ability to secrete proinflammatory cytokines[192]. Thus, the blockade of the STAT3

pathway appears to be a highly promising therapeutic approach. It may potently inhibit Tregs and reverse immunosuppression, while exerting a direct anti-GBMSC effect. However, while the STAT3 blockade response was powerful, it was not complete, suggesting that there may be STAT3-independent pathways mediating immunosuppression induced by GBMSCs.

Culturing monocytes with GBM tumor cells consistently induced STAT3, which correlated with the up-regulation of IL-10 and demanded cell-cell interactions. Furthermore, there was a concurrent up-regulation of STAT3 in tumor cells, indicating the presence of characteristic bidirectional signaling[214]. Additionally, the cytokine microarray analysis revealed the up-regulation of MIC-1 in GBMSCs, which was found to be elevated in the cerebrospinal fluid of GBM patients, with higher levels associated with poorer outcome[217,246]. The potent induction of STAT5 was also observed and recent reports have identified several additional molecules capable of repressing NF-κB–mediated transcriptional activity[214,247,248]. Whether there is a single, dominant molecule or a complex network of inhibitory pathways responsible for the suppression of the monocytic/microglial function remains an important unanswered question. A comprehensive, array-based approach will be necessary to fully appreciate the extent of pathways and other mechanisms by which GBMSCs affect monocytic/microglial activation.

### Allergy- and Inflammation-Related Genes

Epidemiological studies provide strong and consistent evidence of an inverse relationship between the occurrence of allergies and the diagnosis of malignant glial tumors[249]. However, it was not elucidated whether allergies reduce glioma risk or whether their relative absence in glioma patients is merely a reflection of the immunosuppressive effects of the tumor. There is extensive evidence showing that chronic inflammatory disease and other sources of inflammation increase the risk of cancer in several different tissues and organs, but that the connections between glioblastoma progression and inflammation-related genes are not fully understood. A recent comprehensive study on more than 600 allergy- and inflammation-related genes during GBM progression revealed the down-regulation of the majority of investigated genes, indicating widespread immunosuppression, which was not orchestrated by either IL-10 or any of the TGF-β superfamily genes. Surprisingly, differences in the immune function genes correlated with the CD133 expression rather than with the progression of the disease[182]. In contrast to the overall negative trend, the positive associations between GBM and several genes were obtained, including IL-17β, two IL-17 receptors, NCAM-1, and PDGFR-α[182]. There are several reports documenting the elevated expression of IL-17 in different tumors, including gliomas, and it was proposed that TGF-β–induced IL-17 secretion promotes tumor progression[250,251,252,253]. These results provide a challenging setting for further experimental research and suggest alterations of the immune function with glioblastoma progression.

## CONCLUDING REMARKS

The existence of GBMSCs prompts a refocusing of our views on GBM biology with major theoretical and methodological consequences for future research, since it indicates that the biological significance of observations collected on whole tumor tissues should be re-evaluated in terms of their relation to the GBMSC subset. For instance, signaling pathways that are active in the majority of tumor cells might be of little functional relevance for the biology and homeostasis of GBMSCs, and vice versa. Molecular pathways and regulators, active only on a small population of GBM cells, might play the key role in the self-renewal and maintenance of GBMSCs, and therefore largely influence the overall long-term behavior of the tumor. Studies of signaling pathways and other intrinsic factors (such as epigenetic regulators) that are differentially expressed in GBMSCs and NSCs may delineate valid prognostic markers and specific molecular targets with tolerable toxicity.

Numerous studies employ genomic and epigenomic signatures, as well as other molecular strategies, to predict GBM patient prognosis and response to therapy, and to develop novel drugs. However, most of these studies have used whole tumor specimens as RNA sources, where the most relevant expression pattern from tumor stem cells may be lost in the larger signal from the nonstem tumor cells. Therefore, clear demarcation between GBMSCs and GBM nonstem cells is urgenly needed in future research.

Over the past 25 years, adoptive and active immunotherapies using lymphokine-activated killer cells, cytotoxic T cells, TILs, autologous tumor cells, and dendritic cells have been tested in phase I/II clinical trials with high-grade glioma patients and exhibited limited success. Elucidating how GBMSCs and their microenvironment influences and "educates" immune cells might result in more effective immunotargeting of this particular subpopulation. Recently, several studies elucidated a few mechanisms of GBM-mediated immunosuppression and also showed that these effects may be diminished, targeting both GBMSCs and immunosuppressive cells by small molecular inhibitors, or inducing the differentiation of the GBMSCs. Therefore, while the clinical relevance of GBMSCs remains a fundamental issue in this rapidly emerging field, current findings suggest that the specific elimination of these cells and the recovery of the immune system are possible and therapeutically relevant.

## ACKNOWLEDGMENTS

This work was supported by a grant, No 175092, from the Ministry of Science and Technological Development of Serbia.
